# Pregnancy Outcomes in Patients With Multiple Sclerosis Exposed to Natalizumab—A Retrospective Analysis From the Austrian Multiple Sclerosis Treatment Registry

**DOI:** 10.3389/fneur.2020.00676

**Published:** 2020-08-04

**Authors:** Michael Guger, Gerhard Traxler, Martina Drabauer, Doris Leitner-Pohn, Christian Enzinger, Fritz Leutmezer, Dierk Oel, Franziska Di Pauli, Thomas Berger, Gerhard Ransmayr

**Affiliations:** ^1^Department of Neurology 2, Kepler University Hospital GmbH, Linz, Austria; ^2^Medical Faculty, Johannes Kepler University Linz, Linz, Austria; ^3^Department of Neurology, Medical University of Graz, Graz, Austria; ^4^Department of Neurology, Medical University of Vienna, Vienna, Austria; ^5^Department of Neurology, Academic Teaching Hospital Wels-Grieskirchen, Wels, Austria; ^6^Clinical Department of Neurology, Medical University of Innsbruck, Innsbruck, Austria

**Keywords:** breastfeeding, fetal safety, multiple sclerosis, natalizumab, pregnancy outcome

## Abstract

**Objectives:** To analyze safety and impact of natalizumab (NTZ) exposure on the disease course, pregnancy, and newborn outcomes of relapsing-remitting multiple sclerosis (RRMS) patients from the Austrian Multiple Sclerosis Treatment Registry (AMSTR).

**Materials and Methods:** Twelve pregnancies of 11 women with RRMS exposed to treatment with NTZ were identified from the AMSTR. Exposure to NTZ was defined as treatment with NTZ from 8 weeks prior to the start of the last menstrual period and onward. All patients completed a standardized questionnaire regarding pregnancy and newborn outcomes until the postpartum period for up to 12 months.

**Results:** NTZ was stopped on average 46 days after the last menstrual period. There were 11 live births and one elective termination due to ectopic pregnancy. Mean gestational age of live born individuals was 39.0 weeks [standard deviation (SD) ± 1.1]. Mean birth weight and length were 3,426 g (SD ± 348) and 51.9 cm (SD ± 1.9), respectively. Apgar scores 1 min after birth were normal, with 9.2 points on average. One child displayed hip dysplasia as the only congenital malformation documented in this cohort. Three patients experienced relapses during pregnancy and three patients in the postpartum period, resulting in confirmed Expanded Disability Status Scale (EDSS) progression in four of them.

**Conclusion:** In this cohort, there was no increased risk concerning pregnancy and newborn outcomes due to NTZ exposure. However, relapses occurring during pregnancy and postpartum period resulted in confirmed disability.

## Introduction

Multiple sclerosis (MS) is a chronic inflammatory demyelinating disease of the central nervous system diagnosed predominantly in females mostly during the reproductive age (1). Female patients thus need to be counseled accordingly to balance the potential benefits and risks of drug exposure when considering treatment options before or during pregnancy (2). Importantly, there is a high degree of variability in the course of disease and its response to treatment. Natalizumab (NTZ), a therapeutic monoclonal antibody, is approved for the treatment of relapsing-remitting MS (RRMS) and is widely used in the treatment of RRMS. However, limited data are available on the outcome of exposure to NTZ during pregnancy (3–9). On the other hand, there is also limited information concerning the course of MS during pregnancy after NTZ cessation (6, 9, 10), which, however, would be needed for optimizing patient management in this particular scenario.

Our aim therefore was to analyze the impact of NTZ exposure and cessation on disease course and pregnancy outcome of pregnant MS patients documented in the Austrian Multiple Sclerosis Treatment Registry (AMSTR).

## Materials and Methods

### Data Collection

The AMSTR had been established in 2006 as part of MS network activities in Austria, consisting of all MS clinics and some specially qualified neurological practices (“MS-centres,” in total comprising ~4,300 patients). It maintains quality control and compliance with reimbursement regulations including treatment indications of the Austrian sick funds and contains the clinical profiles of the included patients and MRI and safety data (11–13). In Austria, prescriptions of disease-modifying therapies (DMTs) for MS are exclusively reserved for MS centers. Thus, one can expect data to be evenly collected all over Austria. The AMSTR is compliant with Austrian laws on bioethics, and it also was approved by the ethical committee of the Medical University of Vienna (EC number 2096/2013). The present study on pregnant women exposed to NTZ had been approved by the ethical committee of the State of Upper Austria (EC application number K-66-15).

The AMSTR anonymously documents baseline data such as MS onset and duration, relapses during the 12 months before baseline, the Expanded Disability Status Score [EDSS], gross MRI activity, and use of previous DMTs. Follow-up data (relapses, EDSS, adverse events, change or discontinuation of treatment) are required to be documented every 3–6 months.

Each suspected relapse is confirmed by a specialized neurologist from an MS center and documented in the AMSTR. Relapses are defined as new or worsening neurological symptoms lasting for at least 24 h in the absence of fever. Documentation requires relapse onset, EDSS, and use/dose of intravenous (i.v.) methylprednisolone treatment. Confirmed disability progression is defined as an increase from baseline of at least 1.0 point in the EDSS (or at least 0.5 points for patients with a baseline EDSS higher than 5.5) persisting for at least 24 weeks.

Besides the fact that registration in the AMSTR is mandatory for reimbursement of therapy costs, independent external data monitoring concerning completeness and plausibility of documented data represents a quality-related feature of the AMSTR.

In 2006, the European Medicines Agency (EMA) approved NTZ for RRMS patients. Reimbursement for NTZ in Austria adheres to this approval. Thus, MS patients to be treated with NTZ have to have either a minimum of one relapse in the prior 12 months under treatment with interferon beta or glatiramer acetate and at least 9 T2 lesions or one gadolinium-enhancing lesion on a recent brain MRI (“indication A”) or, if the person has been previously untreated, two or more severe relapses in the preceding 12 months and one or more gadolinium-enhancing lesions on brain MRI or a significant increase in T2 lesion load as compared to a previous recent MRI (“indication B”).

For the present analyses, we selected all female MS patients treated with NTZ within the AMSTR with confirmed pregnancy between 2006 and the end of the year 2016. Exposure to NTZ was defined as treatment with NTZ from 8 weeks prior to the start of the last menstrual period (LMP) onward. Patients were followed until 12 months postpartum. A standardized questionnaire on patient- and pregnancy-related outcome as well as child-specific data was completed by the treating neurologist (Table 1).

**Table 1:** Questionnaire on patient- and pregnancy-related data as well as child-specific data and postpartum period.

**A. Patient characteristics** – Date of birth – First symptoms of disease – Date of diagnosis of disease – Course of MS – Number of relapses in the medical history and during the last year before – pregnancy – EDSS 1 year before and at onset of pregnancy – Co-medication – Family history of MS – Coexisting diseases **B. Pregnancy and delivery** – Previous pregnancies – LMP – Calculated date of birth – Relapse(s) during pregnancy – Drug therapy during pregnancy – EDSS at onset of pregnancy – Pregnancy complications – Exposure to toxins – Way of delivery (vaginal/cesarean) – Epidural anesthesia **C. Newborn** – Sex of newborn – Gestational age – Birth weight – Birth length – APGAR score 1 min after birth – Congenital malformations **D. Postpartum phase (first control within 0–3 months, second control at 9–12 months)** – Breastfeeding – Relapse(s) – EDSS – Restart of MS treatment

*APGAR, Appearance, Pulse, Grimace, Activity and Respiration; EDSS, Expanded Disability Status Scale; LMP, last menstrual period; MS, multiple sclerosis*.

The primary outcome measures were (a) safety and impact of NTZ exposure on pregnancy and newborn outcomes of RRMS patients and (b) the impact on disease course during pregnancy and postpartum period.

### Statistical Methods

Descriptive statistics, including mean ± standard deviation (SD), as well as range were used to describe the data.

## Results

Eleven women with 12 pregnancies under NTZ treatment had been registered between 2006 and the end of December 2016. One patient became pregnant twice, and both pregnancies were included in this analysis.

In this cohort, mean age at diagnosis of MS was 25 years (SD ± 5.5) and 30 years (SD ± 5.4) at LMP. All women had received NTZ as second- or third-line therapy (“indication A”), with an average of 22 (SD ± 9.6) and a minimum of seven administrations before pregnancy. Treatment before NTZ included beta-interferons, glatiramer acetate, and fingolimod. A stable course of disease had been achieved with NTZ in all women prior to pregnancy, without an increase in the EDSS score at pregnancy onset (mean 1.3, SD ± 1.1) compared to the EDSS 1 year before (mean 1.3, SD ± 1.1). NTZ infusions were terminated after a mean interval of 46 days (SD ± 38) after LMP. In one patient, treatment was continued after confirmed pregnancy until the 20th week of pregnancy (Day 137) because of high disease activity before NTZ start and suspected rebound after NTZ cessation.

Maternal baseline data are summarized in Table 2.

**Table 2:** Maternal baseline data.

**Characteristic**	
**Age at MS diagnosis (years)**
Mean ± SD	25.3 ± 5.5
Range (min–max)	18.8–34.9
**Age at last menstrual period (years)**
Mean ± SD	30.1 ± 5.4
Range (min–max)	20.0–39.4
**Number of administrations of NTZ**, ***n***
Mean ± SD	22.2 ± 9.6
Range (min–max)	7–40
**Number of previous treatments before NTZ**, ***n*** **(%)**
No prior treatment	0 (0)
1 therapy	7 (64)
2 therapies	4 (36)
>2 therapies	0 (0)
**Total number of relapses prior to pregnancy**
Mean ± SD	7.6 ± 3.3
Range (min–max)	3–13
**Number of relapses within 12 months prior to pregnancy**
Mean ± SD	0.36 ± 0.5
Range (min–max)	0–1
**EDSS 12 months prior to pregnancy**
Mean ± SD	1.3 ± 1.1
Range (min–max)	0–3
**EDSS at pregnancy onset**
Mean ± SD	1.3 ± 1.1
Range (min–max)	0–3
**Timing of exposure to NTZ in pregnancy**, ***n*** **(%)**
Within 8 weeks before last menstrual period	1 (8.3)
First trimester	9 (75)
After first trimester	2 (16.7)
During all pregnancy	0 (0)
**Timing of exposure to NTZ in pregnancy (days)**
Mean ± SD	46.2 ± 37.8
Range (min–max)	−19–137
**Obstetric history**, ***n*** **(%)**
Primiparous	8 (75)
Previous pregnancies	4 (25)
Previous miscarriages/abortions	0 (0)
Previous stillbirths/neonatal deaths	0 (0)
**Positive family history for MS**, ***n*** **(%)**
Yes	4 (36)
No	7 (64)
**Concomitant disorders**, ***n***
Thyroid cancer	1
Diabetes mellitus type I	1
Leukocytoclastic vasculitis	1
Polyarthritis	1
Vitamin-B-12 malabsorbtion deficiency	1

*EDSS, Expanded Disability Status Scale; max, maximum; min, minimum; n, number; NTZ, natalizumab; SD, standard deviation;%, percentage*.

Eight women were primiparous. Eleven live births and one elective termination/miscarriage due to ectopic pregnancy occurred. There was no stillbirth or neonatal death. Vaginal delivery was performed in seven (63.6%) compared to cesarean section in four women (36.4%). A total of five patients (45.5%) received epidural anesthesia. Mean gestational age of live births was 39.0 weeks (SD ± 1.1).

Most children were delivered as early terms (between 37 weeks/0 days and 38 weeks/6 days). There was no preterm delivery (before 37 weeks/0 days).

Three patients suffered from an MS relapse during pregnancy. NTZ was terminated in two cases 43 and 51 days after the LMP; in one case, the relapse occurred during NTZ treatment (neutralizing antibodies against NTZ negative). All relapses occurred at the end of the first trimester. One of these relapses was treated with standard high-dose methylprednisolone and another one with i.v. immunoglobulins to avoid adverse events with high-dose methylprednisolone. Two of these three patients showed confirmed EDSS progression (from EDSS 1.0–2.0 and EDSS 1.5–2.5, respectively) (Figures S1, S2).

Apart from treatment of relapses, there was no further documented exposure to any other potential toxin. Pregnancy outcomes are shown in Table 3.

**Table 3:** Pregnancy outcomes.

**Outcome**	
**Delivery**, ***n*** **(%)**
Vaginal	7 (63.6)
Cesarean	4 (36.4)
**Epidural anesthesia**, ***n*** **(%)**
Yes	5 (45.5)
No	6 (54.5)
**Delivery outcome**, ***n*** **(%)**
Live birth	11 (91.7)
Elective termination	1 (8.3)
Miscarriage	0 (0)
Stillbirth	0 (0)
Neonatal death	0 (0)
**Gestational age of live births**
Preterm (before 37 weeks, 0 days)	0 (0)
Early term (between 37 weeks, 0 days, and 38 weeks, 6 days)	8 (72.7)
Full-term (between 39 weeks, 0 days, and 40 weeks, 6 days)	2 (18.2)
Late term (between 41 weeks, 0 days, and 41 weeks, 6 days)	1 (9.1)
Post-term (after 42 weeks, 0 days)	0 (0)
**Gestational age (weeks)**
Mean ± SD	39.0 ± 1.1
Range (min–max)	38.0–41.6
**Relapse during pregnancy**, ***n*** **(%)**
no relapse	9 (75)
1 relapse	3 (25)
2 relapses	0 (0)
>2 relapses	0 (0)
**Exposure to toxins**, ***n*** **(%)**
Ionizing radiation	0 (0)
Alcohol	0 (0)
Nicotine	0 (0)
Drugs (other than treatment of relapse)	0 (0)

*n, number; SD, standard deviation;%, percentage*.

Six newborns were female. Mean birth weight was 3,426 g (SD ± 348) and length was 51.9 cm (SD ± 1.9). Apgar score 1 min after birth was within the normal range, with a minimum score of 8 points and 9.2 points on average. One child displayed hip dysplasia, which was the only documented congenital malformation in this cohort.

Eight of 11 women decided to breastfeed at least for 3 months; two of them continued breastfeeding until the second control postpartum.

Eight patients received NTZ for an average of 113 (SD ± 103, range 7–278) days after delivery (as primary choice of therapy), while two patients started with fingolimod 180 and 240 days after delivery, respectively.

The mean EDSS score 12 months postpartum compared to the EDSS score at pregnancy onset showed a deterioration of 0.5 (SD ± 1.3). Three patients suffered from relapses in the postpartum period before restarting NTZ, resulting in a sustained EDSS progression in two of them. The first patient experienced a relapse 3 months after delivery and deteriorated from EDSS 0.0 to 4.0; the other patient had a relapse at 6 months postpartum with an EDSS worsening from 1.0 to 3.0 (Figures S3, S4).

In contrast, two patients experienced an EDSS improvement (Table 4).

**Table 4:** Postpartum data.

**Breastfeeding, *n* (%)**	
0–3 months	6 (54.5)
>3 months	2 (18.2)
No breastfeeding	3 (27.3)
**Treatment within 12 months after delivery, *n* (%)**
Natalizumab	8 (72.8)
Fingolimod	2 (18.2)
No drug	1 (9.1)
**Restart of medication after delivery (days)**
Mean ± SD	132 ± 101
Range (min–max)	7–278
**EDSS at delivery**
Mean ± SD	2 ± 1.4
Range (min–max)	0–4.5
**EDSS 12 months postpartum**
Mean ± SD	1.8 ± 1.4
Range (min–max)	0–4
EDSS change^*^
Mean ± SD	0.5 ± 1.3
Range (min–max)	−0.5–4
**Relapse within 12 months after delivery, *n* (%)**
No relapse	8 (72.7)
1 relapse	2 (18.2)
2 relapses	1 (9.1)
>2 relapses	0 (0)

*EDSS, Expanded Disability Status Scale; max, maximum; min, minimum; n, number; SD, standard deviation;%, percentage*.

* *EDSS at pregnancy onset compared to 12 months postpartum*.

## Discussion

So far, limited data are available on pregnancies in MS patients with NTZ (3–10) Ebrahimi et al. (4) documented 102, Friend et al. (7) 355, and Portaccio et al. (8) 92 pregnancies under NTZ treatment. The spontaneous abortion rate of MS women who received NTZ during pregnancy was similar to that of the general population; however, birth defect rates seemed slightly higher than that in the general population and disease-matched groups (6).

In our study, NTZ was continued for a mean of 46 days after LMP with no further negative impact on pregnancy and delivery. This fact corroborates previous publications showing similar results. Out of 12 pregnancies, there was one ectopic pregnancy and hip dysplasia, respectively.

In comparison to healthy controls, the observed birth weight was lower in this cohort, which has been reported previously among women who became pregnant under NTZ (4, 6, 7, 14–16). There was no newborn with a low birth weight (<2,500 g) nor was there a preterm delivery in our cohort.

The EDSS score remained stable in all patients who had no relapses during pregnancy or postpartum period. In contrast, four of six patients who had suffered from relapses during pregnancy or the postpartum period showed a confirmed EDSS progression. Also in this respect, our study further affirms previous publications (4, 9, 10, 17). This underlines the importance to shorten the treatment gap to reduce the maternal risk of experiencing disease activity during pregnancy or thereafter.

Our study has several limitations. Apart from the retrospective nature of the study, the sample size of women was small. However, this study contributes to the still limited number of reported pregnancies with prior exposure to NTZ with further 12 cases. In a recent review regarding NTZ exposure during pregnancy in multiple sclerosis, several studies with a similar or even smaller number of pregnancies as in our work were included and added valuable information to the review (6). Clearly, higher sample sizes would allow to draw firmer conclusions. Therefore, our study should stimulate further investigations into this area, e.g., by collaborative efforts across academic centers and countries.

In conclusion, NTZ seems to be safe in early pregnancy; however, termination of NTZ in patients with active RRMS may harbor the risk of reoccurrence of disease activity with potential disability.

## Data Availability Statement

The datasets generated for this study are available on request to the corresponding author.

## Ethics Statement

The studies involving human participants were reviewed and approved by ethical committee of the State of Upper Austria (EC application number K-66-15). The patients/participants provided their written informed consent to participate in this study.

## Author Contributions

MG contributed patients, performed the statistic, and wrote the manuscript. GR performed the statistic and revised the manuscript. TB, FD, DO, FL, CE, and DL-P contributed patients and revised the manuscript. MD contributed patients and performed the statistic. GT performed the statistic and wrote the manuscript. All authors contributed to the article and approved the submitted version.

## Conflict of Interest

MG received support and honoraria for research, consultation, lectures and education from Almirall, Bayer, Biogen, Celgene, Genzyme, MedDay, Merck, Novartis, Octapharma, Roche, Sanofi Aventis, Shire and TEVA ratiopharm. GT received support and honoraria for research, lectures and education from Bayer, Biogen, Boehringer Ingelheim, Bristol-Myers Squibb, Celgene, EVER Pharma, Genzyme, Merz Pharma, Novartis, Sanofi-Aventis, Pfizer and TEVA. DL-P received support and honoraria for research, lectures and education from Roche and Sanofi-Aventis. CE has received funding for travel and speaker honoraria from Biogen, Bayer, Merck Serono, Novartis, Shire, Genzyme and Teva Pharmaceutical Industries Ltd./sanofi-aventis; research support from Merck Serono, Biogen, and Teva Pharmaceutical Industries Ltd./sanofi-aventis; and has served on scientific advisory boards for Bayer Schering, Biogen, Celgene, Merck Serono, Novartis, Roche and Teva Pharmaceutical Industries Ltd./sanofi-aventis. FL received support and honoraria for research, consultation, lectures and education from Bayer, Biogen, Celgene, Genzyme, MedDay, Merck, Novartis, Octapharma, Roche, Sanofi Aventis, and TEVA ratiopharm. DO received honoraria for lectures and congress support by Biogen, Genzyme and Novartis. FD has participated in meetings sponsored by, received honoraria (lectures, advisory boards, consultations), or travel funding from Bayer, Biogen, Merck, Novartis, Sanofi-Genzyme, Teva, Celgene and Roche. TB has participated in meetings sponsored by and received honoraria (lectures, advisory boards, consultations) from pharmaceutical companies marketing treatments for multiple sclerosis: Almirall, Bayer, Biogen, Biologix, Bionorica, Genzyme, MedDay, Merck, Novartis, Octapharma, Roche, Sanofi/Genzyme, TG Pharmaceuticals, TEVA-ratiopharm and UCB. His institution has received financial support in the last 12 months by unrestricted research grants (Biogen, Bayer, Merck, Novartis, Sanofi/Genzyme, and TEVA ratiopharm) and for participation in clinical trials in multiple sclerosis sponsored by Alexion, Bayer, Biogen, Merck, Novartis, Octapharma, Roche, Sanofi/Genzyme, and TEVA. GR received consultation honoraria and congress support by Biogen, Genzyme, Novartis, Roche and Teva. The remaining author declares that the research was conducted in the absence of any commercial or financial relationships that could be construed as a potential conflict of interest.
