# Supplementary figures and images for: Pregnancy Outcomes in Patients With Multiple Sclerosis Exposed to Natalizumab—A Retrospective Analysis From the Austrian Multiple Sclerosis Treatment Registry
Source: Front Neurol. 2020 Aug 4;11:676. doi: 10.3389/fneur.2020.00676 (PMC7417297; doi:10.3389/fneur.2020.00676)

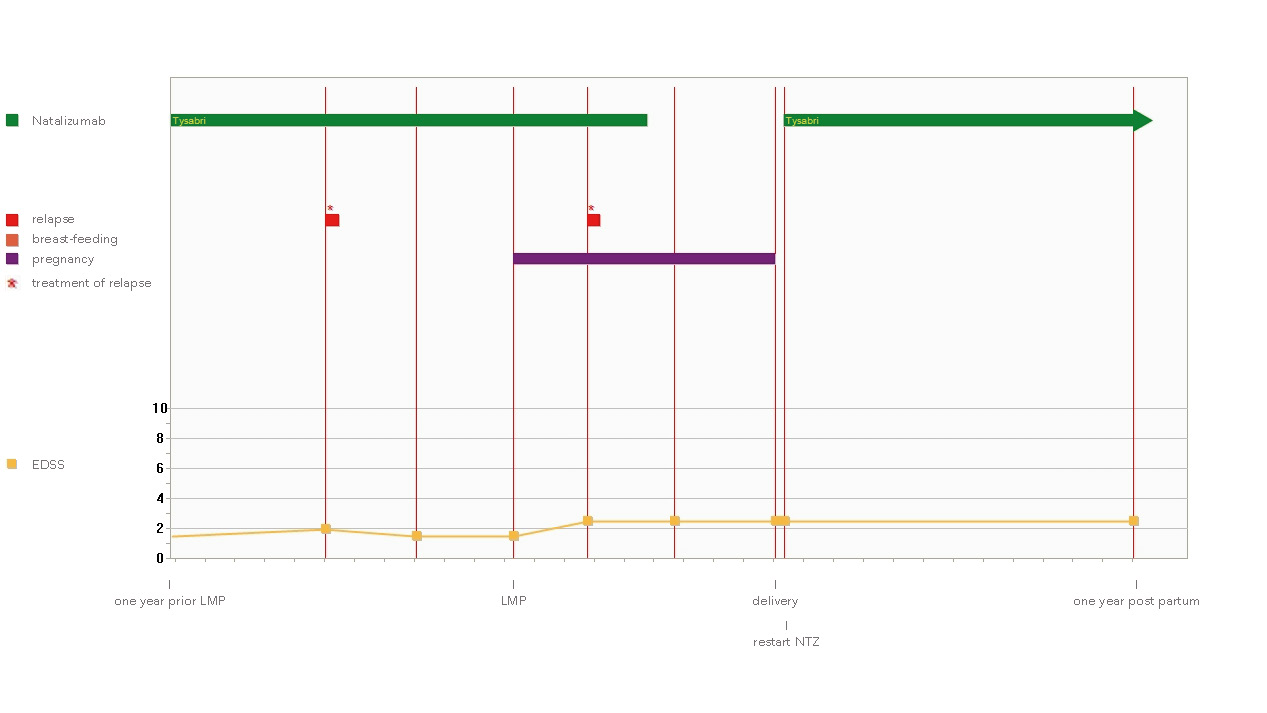

Supplement: Supplementary file 1 [file Image_1.JPEG]

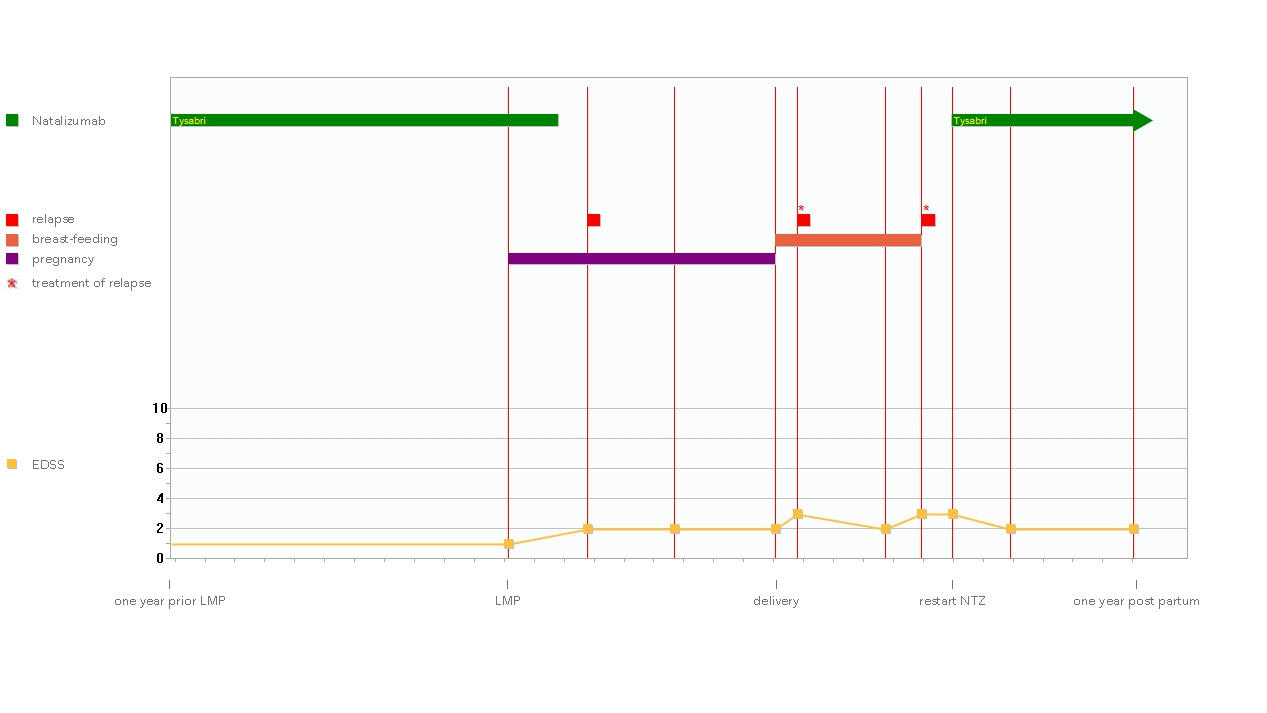

Supplement: Supplementary file 2 [file Image_2.JPEG]

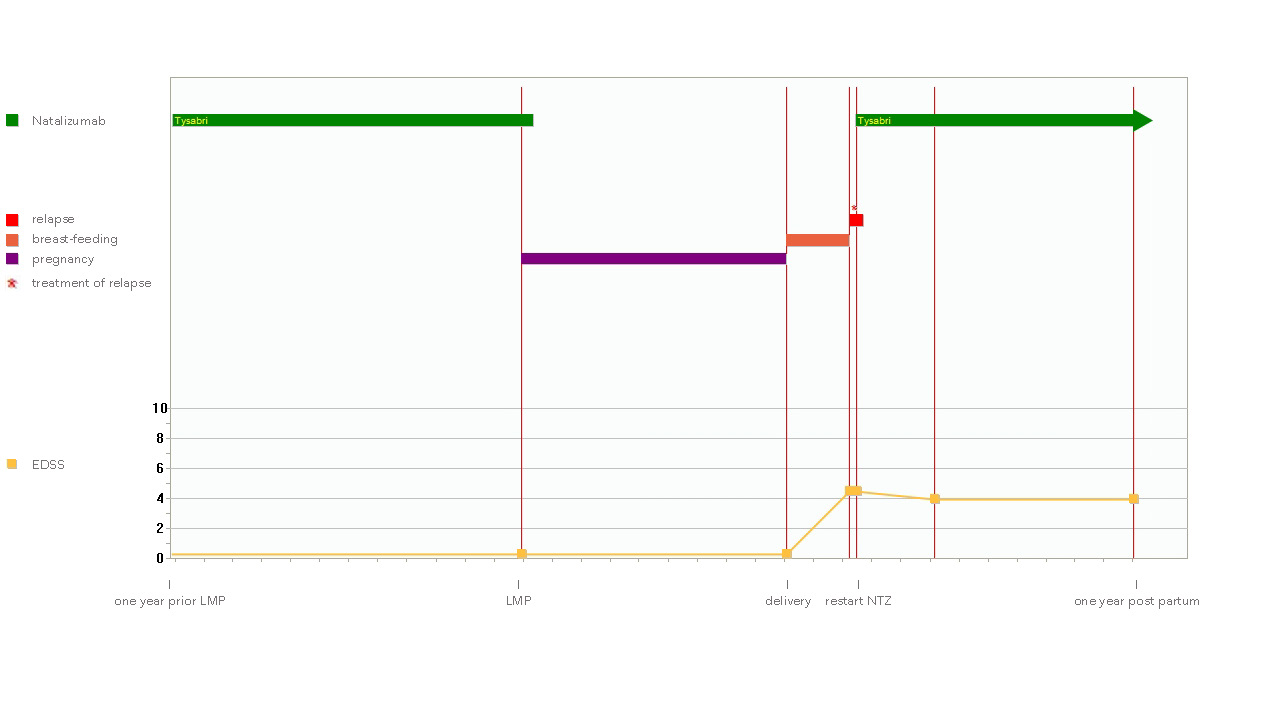

Supplement: Supplementary file 3 [file Image_3.JPEG]

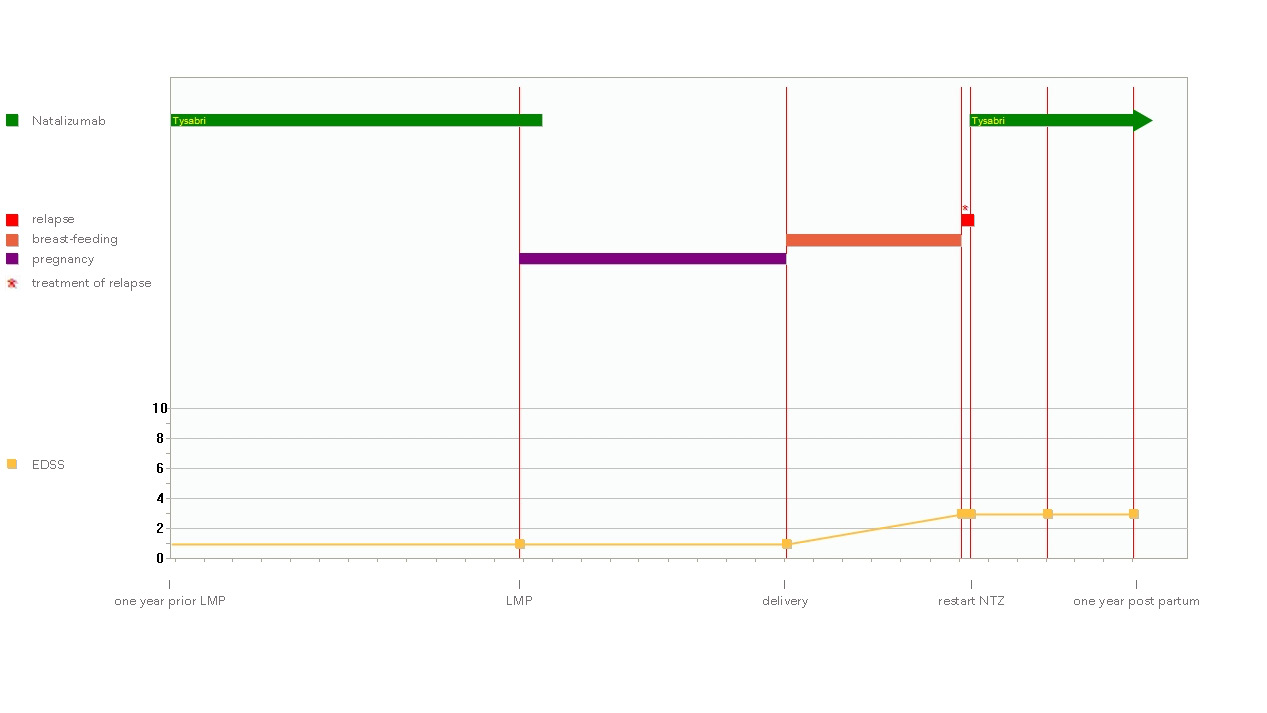

Supplement: Supplementary file 4 [file Image_4.JPEG]
