# Supplementary material for: Pregnancy Outcomes in Patients With Multiple Sclerosis Exposed to Natalizumab—A Retrospective Analysis From the Austrian Multiple Sclerosis Treatment Registry
Source: Front Neurol. 2020 Aug 4;11:676. doi: 10.3389/fneur.2020.00676 (PMC7417297; doi:10.3389/fneur.2020.00676)
Supplement: Supplementary file 5 [file Data_Sheet_1.DOCX]

Figure S1. Patient with MS relapse during pregnancy and confirmed EDSS progression

Abbreviations: EDSS = Expanded Disability Status Scale; LMP = last menstrual period; MS = multiple sclerosis; NTZ = natalizumab

Figure S2. Patient with MS relapse during pregnancy and confirmed EDSS progression

Abbreviations: EDSS = Expanded Disability Status Scale; LMP = last menstrual period; MS = multiple sclerosis; NTZ = natalizumab

Figure S3. Patient with MS relapse during postpartum period and confirmed EDSS progression

Abbreviations: EDSS = Expanded Disability Status Scale; LMP = last menstrual period; MS = multiple sclerosis; NTZ = natalizumab

Figure S4. Patient with MS relapse during postpartum period and confirmed EDSS progression

Abbreviations: EDSS = Expanded Disability Status Scale; LMP = last menstrual period; MS = multiple sclerosis; NTZ = natalizumab
